# The Effect of Ingested Macronutrients on Postprandial Ghrelin Response: A Critical Review of Existing Literature Data

**DOI:** 10.1155/2010/710852

**Published:** 2010-02-02

**Authors:** Chrysi Koliaki, Alexander Kokkinos, Nicholas Tentolouris, Nicholas Katsilambros

**Affiliations:** First Department of Propaedeutic Medicine, Laiko General Hospital, Athens University Medical School, Agiou Thoma 17, 115 27 Athens, Greece

## Abstract

Ghrelin is a powerful orexigenic gut hormone with growth hormone releasing activity. It plays a pivotal role for long-term energy balance and short-term food intake. It is also recognized as a potent signal for meal initiation. Ghrelin levels rise sharply before feeding onset, and are strongly suppressed by food ingestion. Postprandial ghrelin response is totally macronutrient specific in normal weight subjects, but is rather independent of macronutrient composition in obese. In rodents and lean individuals, isoenergetic meals of different macronutrient content suppress ghrelin to a variable extent. Carbohydrate appears to be the most effective macronutrient for ghrelin suppression, because of its rapid absorption and insulin-secreting effect. Protein induces prolonged ghrelin suppression and is considered to be the most satiating macronutrient. Fat, on the other hand, exhibits rather weak and insufficient ghrelin-suppressing capacity. The principal mediators involved in meal-induced ghrelin regulation are glucose, insulin, gastrointestinal hormones released in the postabsorptive phase, vagal activity, gastric emptying rate, and postprandial alterations in intestinal osmolarity.

## 1. Introduction

Ghrelin is a 28-amino-acid gastrointestinal peptide with appetite-stimulating, growth hormone-releasing and adipogenic properties [1–3]. It was originally characterized as the endogenous ligand for the hypothalamic-pituitary growth hormone secretagogue receptor type 1a (GHSR1a), stimulating the anterior gland of pituitary to produce GH [1–3]. In fact, ghrelin is the third physiological regulator of endogenous GH secretion, along with hypothalamic GH releasing hormone and somatostatin. Ghrelin is predominantly produced in the so-called X/A-like endocrine cells of gastric mucosa, and is subsequently released into bloodstream [4, 5]. Ghrelin-producing cells are mostly abundant in the oxyntic glands of gastric fundus [4, 5]. Given the widespread distribution of GHSR1a in the human body, ghrelin exerts pluripotent biological activities, affecting cardiovascular system, pancreatic endocrine function, gastrointestinal tract motility, gastric acid secretion, cell proliferation and metabolism [3]. 

One of the most important actions of ghrelin is its regulatory role for long-term energy homeostasis and short-term food intake [6]. There is a competitive interaction between ghrelin and leptin in hypothalamus for feeding regulation. Ghrelin activates neuropeptide Y (NPY) and Agouti-related protein (AGRP) neurons in the hypothalamic arcuate nucleus, providing a central stimulus for increased food intake and reduced energy expenditure [7]. Intracerebroventricular administration of ghrelin in rodents and peripheral administration in humans has shown to promote weight gain, by reducing fat utilization and increasing food consumption [8, 9]. Ghrelin is actually the only known appetite-stimulating gastrointestinal hormone. It acts as a circulating orexigenic signal, and has been also implicated in preprandial hunger and meal initiation. Cummings et al. were the first to show that plasma ghrelin levels increase nearly twofold immediately before feeding onset, and are strongly suppressed by food ingestion, falling to trough (nadir) levels within an hour after meal initiation [10]. This pattern of secretion is interestingly reciprocal to that of insulin, which is preprandially low and increases gradually in the postabsorptive period [10]. Another interesting finding is that plasma ghrelin levels reflect human nutritional state [11]. Ghrelin secretion is typically up-regulated under conditions of chronic negative energy balance (anorexia nervosa, heart failure cachexia), and down-regulated in the setting of sustained positive energy balance (obesity). Furthermore, obese subjects fail to exhibit the normal postprandial decline of plasma ghrelin concentrations, observed in normal weight individuals [12]. 

The postmeal inhibition of gastric ghrelin production is proportional to energy load and is profoundly influenced by the meal's macronutrient content [13, 14]. In rodents and normal weight humans, the postprandial drop in ghrelin levels is more pronounced after carbohydrate (CHO) meals than after protein- or fat-enriched diet manipulations [15, 16]. The type of ingested macronutrient seems to affect differentially the magnitude and pattern of postprandial ghrelin suppression. Whether it is the direct intraluminal contact of nutrients with gastric mucosa or the insulin-mediated metabolic response to nutrient ingestion more important for postprandial ghrelin suppression remains still controversial. There is currently growing evidence that ghrelin suppression does not require the presence of nutrients in either the stomach or the duodenum, but requires effective post-gastric and postabsorptive feedback mechanisms, possibly mediated by insulin and gastrointestinal hormones with anorexigenic potential [16]. Vagal activity, gastric emptying rate and postprandial increases of intestinal osmolarity are also active players in meal-induced ghrelin regulation [17, 18]. 

Despite the well-established stimulatory effect of ghrelin on appetite and eating behavior, little information is available regarding its relationship with fasting and postprandial energy expenditure in normal weight and obese humans. In rodents, ghrelin infusion promotes weight gain, both by increasing food intake and by decreasing energy expenditure and fat catabolism [8]. This effect is primarily due to an increase in caloric intake and respiratory quotient (RQ), suggestive of a switch from fatty acid oxidation to glycolysis leading ultimately to fat deposition. St-Pierre et al. examined the relationship between serum ghrelin and resting metabolic rate, thermic effect of food, fasting and postprandial RQ, physical activity level and peak aerobic capacity in 65 lean young women. Significant inverse correlations were reported between ghrelin, resting metabolic rate and thermic effect of food, persisting after adjustment for fat-free mass, fat mass and insulin levels [19]. These results suggest that higher levels of ghrelin are associated with low levels of resting and postprandial thermogenesis, indicating that the metabolic effects of ghrelin may extend far beyond the regulation of satiety and substrate oxidation, serving as a biomarker for decreased energy expenditure in humans. On the other hand, the relationship between ghrelin and energy expenditure in obesity constitutes a matter of debate. In a study by Marzullo et al., the obese subjects with low resting energy expenditure (impaired energy balance) exhibited lower active ghrelin levels, compared with obese subjects with high energy expenditure, indicating that ghrelin secretion and activity might be decreased in cases of obesity with impaired energy expenditure, as part of an obesity-related compensatory mechanism [20]. 

The present review aims to shed light on the underlying mechanisms of postprandial ghrelin regulation. In addition, a significant body of clinical and experimental data will be discussed, elucidating the macronutrient-specific effect of several isocaloric test meals on postprandial ghrelin levels. We searched PubMed and other electronic databases for high quality articles written in English, using the following search terms: ghrelin, macronutrients, carbohydrate, protein, fat and postprandial response.

## 2. Underlying Pathophysiology of Postprandial Ghrelin Regulation

 A recent study in humans demonstrated that ghrelin levels can be suppressed by sham feeding (when nutrients are only smelled, chewed or tasted without being swallowed), as well as by actual feeding, indicating the importance of cephalic response to nutrient intake and supporting the role of vagal activity for the control of postprandial ghrelin secretion [17]. The vagally mediated cephalic phase appears to have a major role in initiating the postprandial fall in ghrelin levels, which are thereafter maintained suppressed, by other—as yet not entirely elucidated—gastrointestinal or postabsorptive mechanisms, mediating the nutrient-related ghrelin response [17]. The gastric phase alone appears to play no role in the regulation of ghrelin secretion, because neither gastric distension alone, nor activation of chemical nutrient-sensing mechanisms of gastric mucosa (gastric chemosensitization) can modulate ghrelin levels [18]. In an interesting experiment in rats, intragastric infusion of glucose reduced ghrelin levels by approximately 50%, while water infusion had no effect [21]. However, when gastric emptying was prevented through the inflation of a pyloric cuff (gastric distension), glucose and water infusions were similarly ineffective to suppress ghrelin. These experimental findings indicate that gastric distension and chemosensation are both insufficient to induce a ghrelin response [21]. Prandial ghrelin regulation is probably mediated by intestinal signals generated downstream of Treitz ligament, meaning that postgastric feedback is definitely required for an adequate inhibitory ghrelin response [16]. In this intestinal phase of food ingestion, there seems to be a prominent macronutrient effect, determining the depth and duration of postprandial ghrelin suppression [17]. The macronutrient-related patterns of ghrelin response imply that either the direct exposure of gastrointestinal mucosa to ingested nutrients, or the increased circulating levels of nutrients or other related hormones, can influence postprandial ghrelin levels in a macronutrient-specific manner [17, 22]. Candidate mediators involved in the regulation of postprandial ghrelin secretion are glucose, insulin, cholecystokinin (CCK), glucagon-like peptide 1 (GLP-1), glucose-dependent insulinotropic polypeptide (GIP), peptide YY (PYY), pancreatic polypeptide (PP), oxyntomodulin and somatostatin (SS). Most of these molecules are gastrointestinal hormones, which delay gastric emptying and display insulinotropic and anorexigenic activities [18, 21]. 

Insulin and glucose are thought to be dynamic modulators of plasma ghrelin concentrations in rodents and humans [23–25]. Hyperglycemia and hyperinsulinemia tend to decrease, while hypoglycemia and insulin deficiency tend to increase circulating ghrelin levels. Both intravenous and oral administration of glucose leads to a significant decline in circulating ghrelin, indicating that ghrelin secretion may be suppressed, at least in part, by an increased plasma glucose level in healthy humans [23, 26]. For intravenous glucose administration in particular, Möhlig et al. showed that glucose elicits a significant decrease of plasma ghrelin concentrations, whereas intravenous free fatty acid or arginine load does not affect circulating ghrelin levels [27]. Insulin-mediated glucose uptake and metabolism may also control postprandial ghrelin levels, while insulin sensitivity is considered to be an important determinant of postprandial ghrelin suppression [28]. A great increase in plasma free fatty acids, as a result of constant intravenous lipid infusion, failed to suppress plasma ghrelin, while ghrelin decreased by almost 50% under hyperinsulinemic euglycemic clamp conditions [27]. 

The effect of acute hyperinsulinemia on plasma ghrelin concentrations is still a matter of debate. Clinical data regarding the existing interrelation between ghrelin and insulin are rather conflicting. According to the study of Flanagan et al., using a stepped hyperinsulinemic eu-, hypo- and hyperglycemic glucose clamp, insulin may suppress circulating ghrelin independently of glucose, although glucose might have an additional synergistic effect [29]. In the same direction, Murdolo et al. tested the hypothesis that insulin is the driving force for postprandial ghrelin suppression by comparing the effects of meal ingestion on plasma ghrelin levels between insulin-deficient patients with type 1 diabetes and healthy controls [30]. The investigators concluded that insulin is essential for meal-induced plasma ghrelin suppression, commenting that severe insulin deficiency in uncontrolled type 1 diabetic subjects may partly explain the episodes of hyperphagia observed in these patients, through compromising postprandial ghrelin response [30]. 

The exact mechanism of insulin affecting plasma ghrelin concentrations remains to be established. Insulin might inhibit ghrelin synthesis or secretion, either directly or indirectly. Studies in rats have shown that insulin-induced hypoglycemia increases, instead of decreasing, ghrelin mRNA levels in the gastric fundus, providing no evidence for a direct inhibitory action of insulin on ghrelin synthesis [11]. However, in healthy humans, plasma ghrelin concentrations are decreased during insulin-induced hypoglycemia, suggesting species-specific differences between rodents and humans [31]. Since ghrelin-producing cells are closely associated with the capillary network of the lamina propria of gastric mucosa, their function might be under endocrine control [5]. However, there is still no evidence for insulin receptors on the surface of ghrelin-producing cells. Indirect pathways for insulin suppressive effects on ghrelin synthesis and secretion include activation of hypothalamic insulin receptors and modulation of the cellular flux of glucose or free fatty acids [32]. There seems to be an interaction between nutrients and insulin in reducing circulating ghrelin levels. A greater insulin-induced glucose uptake by X/A-like cells might inhibit ghrelin synthesis and/or secretion [30]. 

Contrary to the findings mentioned above, Caixás et al. reported that, unlike food intake, the subcutaneous administration of a short-acting insulin analog was not able to suppress ghrelin levels [33]. In concordance with this study, Schaller et al. concluded that meal-related ghrelin suppression is not directly regulated by glucose or insulin, since a reduction in ghrelin was observed only at supraphysiological insulin concentrations, while hyperglycemia did not decrease ghrelin at all [34]. Contrary to Murdolo, Spranger et al. observed a substantial postprandial decrease of plasma ghrelin in an insulin-deficient patient with type 1 diabetes following a carbohydrate challenge, while a subsequent bolus of subcutaneous short-acting insulin induced no further changes in circulating ghrelin [35]. 

Taking everything into consideration, glucose and insulin are unlikely to explain the entire postprandial ghrelin response, since ghrelin remains suppressed long after the normalization of glucose and insulin levels, and furthermore, because lipids tend to suppress ghrelin in the absence of substantial increases in glucose or insulin [16]. Other hormonal mediators (such as CCK, GLP-1 and GIP) released in the postabsorptive phase in response to nutrient stimuli, appear to orchestrate the whole postmeal ghrelin response, enhancing the inhibitory effects of glucose and insulin, which are still remaining the principal contributors. The inverse temporal relationship between circulating concentrations of ghrelin and insulin, reported in a large number of clinical studies, substantiates the key regulatory role of insulin for ghrelin regulation [36–38].

It would be also interesting to examine the role of leptin in postprandial ghrelin regulation, since ghrelin and leptin are part of a dynamic peripheral feedback system that regulates body weight and energy homeostasis by modulating satiety. Data concerning the relationship between ghrelin and leptin at fasting and postprandial state are rather contradictory. In vitro studies have previously shown that leptin inhibits ghrelin production from the gastric mucosa, and leptin levels in humans appear to be inversely related to ghrelin concentrations [39]. On the other hand, the interesting observation by Cummings et al. that leptin and intermeal ghrelin levels display diurnal rhythms that are in phase with one another suggests that leptin and ghrelin might be coordinately regulated [10]. The same study reported a subtle postprandial drop in leptin levels that may reflect meal-related regulation of gastric leptin, according to the investigators [10]. The relationship between insulin and leptin is more clearly defined and may partially explain the ghrelin-leptin interrelationship. Previous studies indicate that leptin secretion is regulated by insulin-mediated glucose metabolism, suggesting that insulin is a positive modulator of leptin concentrations [40]. This is why consumption of high-fat meals and high-fructose beverages that produce smaller postprandial glucose and insulin responses compared with isocaloric high-carbohydrate meals, have shown to reduce 24-hour circulating leptin concentrations in humans [40]. Accepting that leptin and insulin are positively associated, it is conceivable that postprandial surges of insulin are related to increased leptin levels, and given the reciprocal relationship between insulin and ghrelin, a similar inverse relationship might be supported for leptin and ghrelin as well. Because insulin and leptin function as key signals conveying information on energy intake and body fat stores to the central nervous system for the long-term regulation of food intake and energy homeostasis, it is possible that reduced insulin and leptin production, as well as increased ghrelin levels, contribute to increased energy intake, weight gain, and obesity in humans [40].

## 3. Carbohydrate Ingestion and Postprandial Ghrelin Response

In an interesting study by Foster-Schubert et al., three different macronutrient preloads of equal caloric content, volume and energy density (protein-, fat-, and CHO-based beverages) were compared for their relative efficacy to suppress total and acylated (bioactive) ghrelin levels [41]. Total ghrelin levels decreased significantly more after CHO or protein ingestion than after lipids, while ghrelin's nadir (lowest) levels were reached most rapidly after the CHO-enriched preload (within 99 minutes). For both acylated and total ghrelin concentrations, investigators observed marked and macronutrient-dependent differences during the first 3 hours versus the subsequent 3 hours of the postprandial study period. More specifically, they observed only after CHO ingestion a marked rebound of acylated ghrelin to 37% above baseline levels, during the second 3 hours of the 6 hour post-ingestive period. This study reveals a previously unidentified pattern in the response of acylated and total ghrelin after CHO ingestion. Ghrelin levels decreased in the initial 3 hours, followed by a marked overshoot to above the pre-ingestion baseline during the second 3 hours. No such overshoot was observed after protein or lipid ingestion, both of which suppressed acylated and total ghrelin levels until study completion. These observations suggest very different effects of high CHO meals in the early versus the later postabsorptive phase, indicating that ingested CHO might prompt an early hunger rebound. Such findings have important clinical implications for design of dietary regimens [41]. 

The faster gastric emptying after CHO ingestion, compared with lipids or proteins, can partly explain the strong and rapid postprandial ghrelin suppression in the first phase [41]. The rapid removal of CHO from stomach should cause a rapid, strong suppression of ghrelin levels, an effect that might be short lived, because these nutrients are quickly absorbed and metabolized. If insulin-mediated glucose disposal is more important for ghrelin suppression than mere insulin levels, the late post-CHO ghrelin overshoot may result from reduced intracellular glucose metabolism, when glucose levels decreased below baseline [41]. 

Not all types of dietary CHO are likely to have the same effect on postprandial ghrelin levels. In a study comparing high-glucose with high-fructose meals, the mean postprandial suppression of ghrelin was markedly attenuated and significantly less pronounced after consuming the high-fructose meals [40]. Fructose, unlike glucose, does not stimulate insulin secretion from pancreatic beta cells, presumably because of the low number of fructose transporters (GLUT5) on beta cell membrane [40]. Intravenous fructose infusion increases only marginally circulating insulin concentrations, while ingested fructose is ineffective in eliciting postprandial insulin secretion. What is more, fructose does not increase insulin-mediated glucose metabolism or circulating leptin levels [40]. Given the key role of insulin for postprandial ghrelin suppression, ingested fructose suppresses ghrelin poorly. The failure of fructose to effectively suppress ghrelin (impaired satiety), along with the reduced insulin and leptin concentrations, could lead to an increased caloric intake and ultimately contribute to obesity, during chronic consumption of diets high in fructose [40]. 

CHO-enriched test meals, containing both simple and complex carbohydrates, have been used in various clinical studies investigating the differential response of postprandial ghrelin to meals of different macronutrient composition. In all of them, and particularly in normal weight subjects, CHO ingestion provoked a significant postprandial ghrelin decline by approximately 30% from baseline values within 2 hours after meal onset [37, 38, 40–42]. A common finding in all these studies is the inverse correlation between postprandial ghrelin and insulin concentrations throughout the whole study period. While the suppressant effect of CHO on ghrelin levels is well established and taken for granted, the biphasic pattern of ghrelin suppression after CHO intake [41] and the clinically meaningful distinction between glucose and fructose [40], are novel thought-provoking findings that warrant further investigation.

## 4. Protein Ingestion and Postprandial Ghrelin Response

Dietary protein is considered to be the most satiating macronutrient [43]. The higher satiety associated with protein consumption may be at least partially mediated by a protein-induced prolonged postprandial ghrelin suppression [43]. Such a reduction in the orexigenic signal might delay the initiation of a subsequent feeding episode or lower hunger and energy intake. The prolonged suppression of ghrelin after protein intake might relate to the protracted emptying of proteins from stomach, causing a more sustained activation of post-gastric ghrelin-suppressing mechanisms [41]. Additional mechanisms that account for the significant satiating effect of dietary protein include the following: proteins have a larger thermic effect than CHO or fat, since they cannot be stored in the body, but need to be metabolized immediately [44]. Moreover, increased circulating concentrations of amino acids after protein intake stimulate hepatic gluconeogenesis preventing hypoglycemia, and thus promoting satiety [44]. In rats fed on protein-enriched diets, intestinal gluconeogenesis is also induced in the postabsorptive phase [45]. Last but not least, proteins stimulate the secretion of specific gastrointestinal peptides (CCK, GLP-1, GIP) that delay gastric emptying and increase satiety [44]. 

In a study of three isoenergetic meals (balanced, high-fat and high-protein) consumed by healthy young women, acylated ghrelin fell significantly after ingestion of both balanced and high-protein meals, while ghrelin persisted at significantly lower levels than baseline for a longer duration, following the high-protein meal [36]. Apart from prolonging postprandial ghrelin suppression, liquid protein preloads have also shown to prolong the elevation of anorexigenic gastrointestinal hormones, such as CCK and GLP-1 [43]. These responses are observed irrespective of the type of protein consumed (soy, whey, or gluten) [43]. In support of this, Lang et al. has demonstrated no effect of protein type (egg albumin, casein, gelatin, soy protein, pea protein and wheat gluten) on satiety, 24 hour energy intake and postprandial glucose and insulin concentrations [46]. In a further randomized crossover study in healthy adult males, the high-protein meal maintained significantly lower ghrelin levels at 180 minutes compared with the high-CHO and high-fat meals, indicating that dietary protein exhibits longer-term postprandial ghrelin suppression and enhanced satiety [37]. According to Blom et al., the high-protein breakfast decreased postprandial ghrelin secretion more than did the high-CHO breakfast [44]. It also increased glucagon and CCK, tended to increase GIP and GLP-1, and decreased gastric emptying rate, without affecting however ad libitum energy intake. 

Despite the accumulating evidence supporting the satiating and ghrelin-suppressing capacity of dietary protein, there have been a few studies suggesting that protein ingestion stimulates, instead of suppressing, ghrelin levels [38, 47], while an additional study indicated that the satiating effect of protein is practically unrelated to postprandial ghrelin secretion [48].

## 5. Fat Ingestion and Postprandial Ghrelin Response

Ingested lipids appear to suppress the orexigenic hormone ghrelin less effectively than do CHO or protein [41]. The relatively weak ability of this macronutrient to suppress ghrelin can be attributed to the poor stimulation of insulin secretion by lipids as well as to the lower osmolarity of lipid meals and beverages. Postprandial increases of intestinal osmolarity are believed to promote ghrelin suppression. However, lipids contribute fewer osmolar units compared with an isocaloric consumption of CHO or proteins [41]. 

In a study by Pavlatos et al., total ghrelin levels did not decline significantly after a fat-rich meal in normal weight women, as opposed to an isoenergetic protein-rich meal [49]. In a similarly designed study by Tentolouris et al., fat consumption has also displayed a diminished capacity to induce satiety [50]. In this study, the effect of two isocaloric test meals (one rich in CHO and one rich in fat) on postprandial active ghrelin concentrations was comparatively evaluated in lean and obese women. After the fat-rich meal, active ghrelin levels were not significantly suppressed, even in the lean participants. The investigators conclude that increased fat intake might promote obesity not only through its high caloric content and adverse metabolic effects, but also through its failure to suppress postprandial hunger. Erdmann et al. reported a different (more delayed) time pattern of ghrelin suppression after fat ingestion, compared with CHO [47]. More specifically, the fat-rich meal decreased plasma ghrelin levels, but the nadir was reached towards the end of the study period, namely at 180 minutes. 

The potential impact of varying fatty acid composition (saturated, monounsaturated and polyunsaturated fat) on postprandial ghrelin response has been only scarcely investigated. In a relevant double-blind crossover study, researchers assessed two high-fat test meals, one with a high saturated to unsaturated fat ratio (70/30) and the other with a low ratio (55/45), and concluded that increasing saturated fat consumption had no deleterious effects on fasting and postprandial plasma ghrelin concentrations [51].

## 6. Effect of BMI on Nutrient-Related Ghrelin Regulation

BMI, body fat and indices of central fat distribution are inversely associated with fasting plasma ghrelin concentrations. A large number of clinical studies have shown that obese subjects tend to display lower total and acylated ghrelin levels in the fasting state compared with normal weight individuals [50]. This finding appears to be an appropriate compensatory response, so that obese individuals will not get any fatter and lean individuals will not get any thinner (adaptive mechanism for prevention of obesity and cachexia resp.). It has been proposed that the sustained positive energy balance observed in obesity suppresses maximally circulating ghrelin levels, and thus limits flexibility for further short-term feeding regulation. The impaired cholinergic (vagal) regulation of postprandial drop in ghrelin concentrations might be also responsible for the dysregulated ghrelin control in obese subjects [52]. Furthermore, obese subjects are often insulin-resistant and thus hyperinsulinemic, and insulin is a well established inhibitory signal for ghrelin secretion. To the best of our knowledge, the differential rate and magnitude of preprandial rise in ghrelin levels has not been comparatively evaluated in lean and obese individuals. As already mentioned, it is widely accepted that obese subjects exhibit significantly lower fasting ghrelin concentrations than lean, but whether the rate of preprandial ghrelin increase is actually differentiated between lean and obese subjects has been scarcely addressed. In fact, most of the studies that used frequent blood sampling protocols in order to assess the diurnal plasma ghrelin profile in subjects of varying BMI (preprandial and postprandial hormonal alterations), reported no specific BMI-related differences between lean and obese participants in terms of preprandial rate of ghrelin increase [10]. 

Another important aspect of ghrelin regulation in obese subjects is the blunted postprandial ghrelin response. This means that obese subjects have low ghrelin levels preprandially, but postprandial ghrelin secretion is not sufficiently suppressed, suggesting a severe defect in ghrelin-induced satiety mechanisms, which makes them feel still hungry, even though they have just completed their meal. Pavlatos et al. have shown that neither a protein- nor a fat-rich meal was able to elicit a significant acute ghrelin response in obese women [49]. In the same direction, Tentolouris et al. reported that a high-CHO meal (with a well established ghrelin-suppressing potential in lean individuals) was also insufficient to suppress postprandial active ghrelin levels in obese women, indicating a considerable secretory and possibly satiety impairment in these subjects [50]. Another interesting conclusion of the same study was that, the leaner a person is, the higher his fasting ghrelin is, and the steepest its postprandial decline. This means that a lean subject feels quite hungry before meals, but afterwards feels easily satiated. Apart from ghrelin, additional hormonal factors that contribute to this auto-regulation of body weight homeostasis in normal weight subjects include GLP-1, GIP, and PYY, which delay gastric emptying, induce satiety and prevent hyperphagia, and are significantly more functional in lean subjects compared with the obese [53]. However, as a person gains weight, this autoregulatory effect appears to become severely compromised. An obese subject cannot experience postprandial fullness, independently of the macronutrient composition of his meal [50]. 

The fact that lean subjects display higher fasting ghrelin levels than obese does not necessarily mean that they also consume greater amounts of food. On the contrary, food intake is most likely to be increased in obese subjects, because of the blunted postprandial ghrelin response, as described above. Besides, the effect of ghrelin on hunger and satiety sensations is not necessarily translated into alterations in *ad libitum *energy intake, as shown by the study of Erdmann et al. [38]. An additional study by Druce et al. showed that low-dose infusion of ghrelin increased *ad libitum *energy intake at a buffet meal only in the obese group, and not in the lean, indicating that obese people are highly sensitive to the appetite-stimulating effects of ghrelin, even when the circulating ghrelin is low [54]. As a result, the absolute difference of fasting ghrelin levels between lean and obese subjects is not a major determinant of subsequent food intake, since other factors such as endogenous sensitivity to circulating ghrelin, ghrelin activity and postprandial ghrelin changes are thought to play an important role, as well. 

Additional factors that can in part explain the suppressed basal ghrelin levels in obese subjects include hyperleptinaemia and increased circulating levels of IL-1b (interleukin 1b), since both leptin and IL-1b are thought to inhibit ghrelin secretion [7]. Hyperleptinaemia is observed frequently in obesity due to leptin resistance, and high levels of IL-1b and other inflammatory mediators are also a common finding in patients with obesity and metabolic syndrome. Concerning the blunted postprandial ghrelin response in obese subjects, the impaired post-meal elevation of gastrointestinal hormones with anorexigenic and insulinomimetic properties, such as GLP-1, GIP and PYY, has been implicated as an additional significant contributor [55]. As far as insulin resistance is concerned, its role for ghrelin regulation is different in fasting and postprandial state. In fasting, insulin resistance and thus hyperinsulinemia lead to decreased fasting ghrelin levels. Fasting plasma ghrelin concentrations are lower in insulin-resistant obese adults, compared with equally obese individuals with relatively higher insulin sensitivity [28]. On the other hand, postabsorptive insulin resistance and impaired intracellular insulin signaling lead to inadequately suppressed and thus increased levels of ghrelin, since insulin sensitivity is regarded as prerequisite for sufficient postprandial ghrelin suppression [28]. 

The macronutrient-specific effect of meals on postprandial ghrelin levels has interesting implications only in normal weight individuals. In the obese population, the macronutrient effect appears to become blunted and is therefore of minor importance. This hypothesis is further corroborated by a recent study by Heinonen et al., where obese individuals with metabolic syndrome elicited no differences in plasma ghrelin or feelings of hunger and satiety, after consuming two high-CHO meals producing different insulin responses (whole-grain rye bread and wheat bread) [56]. Despite the different insulin response, ghrelin levels did not change in obese patients in response to either type of bread meals. In addition, ghrelin levels did not correlate with insulin or glucose, indicating that regulation of ghrelin might be altered in obese patients with metabolic syndrome independently of insulin [56]. An additional study by Moran et al. revealed a dysregulation of ghrelin homeostasis in overweight women with polycystic ovary syndrome (PCOS), suggesting that women with PCOS exhibit similar ghrelin abnormalities with obese women (down-regulated fasting ghrelin, blunted postprandial ghrelin suppression), and this disorder was not differentially affected by diet macronutrient composition [53]. 

Diet-induced weight loss, contrary to gastric bypass surgery where ghrelin levels remain dramatically decreased, has shown to elevate fasting ghrelin levels and normalize postprandial ghrelin response [57]. This means that when a person loses a significant amount of weight by diet he might feel a greater preprandial desire to eat, but his postprandial satiety is significantly improved. The greater sensitivity to vagal stimulation after weight loss may result in a more pronounced drop in postprandial ghrelin levels, in addition to the improvement in insulin sensitivity, which is a major determinant of postprandial ghrelin suppression [57]. Romon et al. reported that diet-induced weight reduction preferentially improves ghrelin response to a high-CHO meal, compared with a high-fat meal, indicating that weight loss might selectively improve the response of ghrelin to carbohydrate [57].

## 7. Acute Effect of Ethanol and Smoking on Plasma Ghrelin Levels

Zimmermann et al. addressed the interesting question, whether acute ethanol ingestion affects ghrelin secretion [58]. Ghrelin declined significantly within 15 minutes after alcohol drinking, fell to a minimum of 66% of baseline at 75 minutes and remained suppressed until the last sample at 2 hours. Given that alcohol seems to acutely attenuate circulating ghrelin levels and is also known for its satiating power, one might expect from alcohol to promote weight loss. However, its considerable caloric density and its detrimental overall health effects should not be overlooked. 

As far as smoking is concerned, in an interesting study by Kokkinos et al., acute cigarette smoking induced no significant suppression of post-smoking ghrelin in habitual smokers, possibly desensitized to any possible effect of smoking on ghrelin, through prolonged nicotine exposure [59]. On the other hand, there was a progressive decline of ghrelin in non-smokers, reaching its nadir 60 minutes after smoking. Fasting total ghrelin levels were not significantly different between smokers and non-smokers, indicating that smoking is unlikely to exert a long-term anorectic effect in smoking populations. The significant decrease in circulating ghrelin after smoking cessation, reported by Lee et al., provides further evidence for lack of correlation between smoking status and suppressed plasma ghrelin concentrations [60].

## 8. Summary, Conclusions, and Perspectives

Many clinical studies have used isoenergetic test meals (protein-, fat- and CHO-rich) in order to examine the relative efficacy of each macronutrient to suppress postprandial ghrelin. Even though the overall concept in these studies is common, the experimental design (meal composition, measured parameters, blood sampling intervals, duration of post-ingestive period) is slightly or moderately different. This discrepancy may be in part responsible for heterogeneity in findings. Trying to delineate the central message behind all these divergent data, carbohydrate appears to be the most effective macronutrient in terms of postprandial ghrelin suppression, possibly because of its glucose-elevating and insulin-secreting effect. However, recent data indicate that CHO ingestion may provoke a delayed ghrelin rebound in the later postabsorptive period, questioning the role of CHO-rich meals in weight loss dietary approaches. Besides, all types of dietary CHO are not equally effective. Fructose-enriched meals display a poor ghrelin-suppressing capacity, promoting increased caloric intake, weight gain and obesity under conditions of chronic consumption. The most satiating macronutrient appears to be dietary protein. Protein induces prolonged ghrelin suppression and elevation of gut-derived anorexigenic hormones that delay gastric emptying regardless of the type of protein consumed. However, the influence of solid forms of protein (turkey, pork) on postprandial ghrelin levels may require assessment over a longer period of time than 3-4 hours, since slow gastric emptying delays postprandial ghrelin nadir. As far as fat is concerned, it appears to be the least potent ghrelin-suppressant, even in normal weight subjects. Some studies have shown that fat decreases ghrelin concentrations, but later or more weakly than other macronutrients. At the same time, other studies report that fatty meals have absolutely no effect on postprandial ghrelin levels. In obese subjects, postprandial ghrelin response is blunted, and the macronutrient effect on ghrelin levels appears to be rather neutral. However, weight loss restores ghrelin response and leads to a significant improvement of ghrelin-mediated appetite regulation. 

From now on, it would be interesting to evaluate the long-term effect of macronutrient-enriched diet manipulations on fasting and postprandial ghrelin levels. Additional parameters that could possibly influence ghrelin response and should be further investigated are food form and viscosity (liquid, solid, semi-solid products), portion size and meal duration.

## Abbreviations

GH: Growth hormone
GHSR1a: Growth hormone secretagogue receptor type 1a
NPY: Neuropeptide Y
AGRP: Agouti-related protein
CCK: Cholecystokinin
GLP-1: Glucagon-like peptide 1
GIP: Glucose-dependent insulinotropic polypeptide
PYY: Peptide YY
PP: Pancreatic polypeptide
SS: Somatostatin.
